# Survival and prognostic factors for patients with advanced hepatocellular carcinoma after stereotactic ablative radiotherapy

**DOI:** 10.1371/journal.pone.0177793

**Published:** 2017-05-17

**Authors:** Cheng-Hsiang Lo, Jen-Fu Yang, Ming-Yueh Liu, Yee-Min Jen, Chun-Shu Lin, Hsing-Lung Chao, Wen-Yen Huang

**Affiliations:** 1 Department of Radiation Oncology, Tri-Service General Hospital, National Defense Medical Center, Taipei, Taiwan; 2 Department of Radiation Oncology, Yee Ren Hospital, Taoyuan, Taiwan; 3 Institute of Clinical Medicine, National Yang-Ming University, Taipei, Taiwan; Chang Gung Memorial Hospital Kaohsiung Branch, TAIWAN

## Abstract

**Objective:**

To evaluate the survival outcomes and prognostic factors of patients with advanced hepatocellular carcinoma (HCC) who underwent stereotactic ablative radiotherapy (SABR).

**Methods:**

This retrospective study evaluated patients with advanced HCC who underwent SABR between December 2007 and July 2015. All patients had Barcelona Clinic Liver Cancer stage C disease and Child–Turcotte–Pugh (CTP) class A–B function. In-field control (IFC), overall survival (OS), prognostic factors, and toxicity were evaluated.

**Results:**

In this study of 89 patients, the 3-year IFC rate was 78.1%, and the 1-year and 3-year OS rates were 45.9% and 24.3%, respectively. The multivariate analysis revealed that CTP class, the presence of main portal vein tumor thrombosis, and the presence of extrahepatic spread were independent predictors of OS. The expected median OS values among patients with ≥2, 1, and 0 predictors were 4.2, 8.6, and 26.4 months, respectively (*p* <0.001).

**Conclusions:**

SABR may be useful for patients with advanced HCC, and patient selection could be based on the CTP classification, main portal vein tumor thrombosis, and extrahepatic spread.

## Introduction

Hepatocellular carcinoma (HCC) is a common cause of cancer mortality [1]. The high mortality rate is partly attributed to the fact that many patients with newly diagnosed HCC have advanced disease, and approximately 30–40% have Barcelona Clinic Liver Cancer (BCLC) stage C disease [2, 3]. In this context, BCLC stage C HCC represents a disease spectrum characterized by a cancer-related Eastern Cooperative Group (ECOG) performance status of 1–2, macrovascular invasion, and/or extrahepatic spread (ES) [4]. Unfortunately, patients in this subgroup have a poor prognosis, and treatment is generally palliative; currently, sorafenib monotherapy is the standard treatment, based on available randomized studies and BCLC systems [4–7]. However, most sorafenib-treated patients achieve only stable radiological responses and modest survival benefits (2–3 months), and the high costs and restrictive eligibility criteria limit the use of this drug [5, 7]. Therefore, local treatment modalities that can improve therapeutic responses and survival are needed.

Although transarterial chemoembolism (TACE) is commonly used to treat unresectable HCC in Asia, a single TACE session is rarely sufficient to induce a complete response (CR). In addition, the presence of portal vein thrombosis, low vascularity, or side effects also preclude the application of TACE. Notably, stereotactic ablative radiotherapy (SABR) has recently been identified as an alternative or complementary treatment for patients with HCC. Four prospective studies and several retrospective studies have reported the safety of SABR, as well as high 1-year local control rates (70–100%) [8–19]. However, most SABR series have focused on patients with small tumors and early-stage disease, whereas few have evaluated SABR for advanced HCC, which may limit its clinical application [15, 17, 18]. Therefore, the present study aimed to analyze the outcomes and prognostic factors for SABR among patients with BCLC stage C HCC.

## Materials and methods

### Patients

This retrospective study evaluated all patients who underwent SABR between December 2007 and July 2015 at the Tri-Service General Hospital, Taipei, Taiwan. For most patients with HCC, the treatment options were discussed by a multidisciplinary team, and the indications for liver SABR were (1) a Child–Turcotte–Pugh (CTP) class A or B liver function; (2) local disease with minimal or no ES; and (3) an adequate normal liver volume (≥700 cc), except for patients with a small tumor that had failed or was inaccessible to other local treatments. All patients provided written informed consent to receive the selected treatment, and our institutional review board approved the retrospective design of this study.

Our inclusion criteria were as follows: (1) BCLC stage C HCC, based on the presence of symptomatic tumors (e.g., ECOG performance status of 1–2) or vascular invasion/extrahepatic disease confirmed using computed tomography (CT), magnetic resonance imaging (MRI), or positron emission tomography (PET)/CT; (2) a CTP class A–B liver function; (3) an uninvolved liver volume of ≥700 cc; (4) SABR as the main local treatment, defined as no liver-directed therapy within 3 months before SABR; and (5) no other active cancer during the 5 years before SABR. The HCC diagnosis could be based on histological or radiological criteria [6]. All previous treatments were accepted, and all cases were re-staged according to the American Joint Committee on Cancer staging system (7^th^ edition).

### SABR technique and dose

In all patients, SABR was performed using the CyberKnife^®^ image-guided radiosurgery system, and our previous reports have provided details regarding the preparation, CT simulation, dose volume constraints, and treatment [11, 14]. All patients were advised to undergo CT-guided implantation of 5–6 gold fiducials in or near the tumor; these acted as radiographic markers for the Synchrony respiratory motion-tracking system (Accuray Inc., Sunnyvale, CA, USA). Simulation CT with a slice thickness of 1 mm was performed 7–10 days later. Multiphase dynamic MRI scans obtained in the treatment position were occasionally used to facilitate target delineation, depending on the treating physician's discretion. The gross target volume was defined as the visible tumor on simulation images, and patients with implanted fiducials were assigned expanded margins of 0–8 mm to define the planning target volume. Patients without fiducials were assigned asymmetrical margins of 3–8 mm in the axial direction and 8–20 mm in the longitudinal direction, based on organ motion.

The prescribed dose was set using the normal liver volume, tumor volume, the proximity of the tumor to the luminal gastrointestinal tissue, and the tolerances of nearby normal organs. Most patients received 3–5 fractions. Given the non-uniform fractionation, however, the dose regimens were converted to an equivalent dose of 2 Gy per fraction (EQD2), based on the assumption that the α/β value was 10 Gy [20].

### Response and toxicity evaluations

All patients underwent clinical evaluations, liver function testing, and abdominal CT and/or MRI at 2–3 months after the completion of SABR and at 3–4-month intervals thereafter. More frequent follow-ups were occasionally requested by the treating physician, based on the patient's general condition. PET/CT was occasionally used to evaluate local disease or distant metastasis if the CT or MRI findings were equivocal. The modified Response Evaluation Criteria in Solid Tumors were used to evaluate treatment responses [21]. Macrovascular thrombosis responses were evaluated using the criteria proposed by Yoon *et al* [22]. In-field control (IFC) was defined as the absence of progressive disease (PD) within or at the margin of the planning target volume. All other intrahepatic recurrences were classified as intrahepatic out-field failures. Extrahepatic metastasis included all disease at any non-liver site. Toxicities were recorded based on the worst episode, according to the NCI Common Terminology Criteria for Adverse Events (version 3.0).

### Statistical analysis

All analyses were performed using SPSS software (version 17; SPSS Inc., Chicago, IL, USA). Overall survival (OS) was calculated as the time from the last fraction of SABR until death from any cause or the last follow-up. The Kaplan–Meier method was used to compare the OS and IFC rates, and differences were evaluated using the log-rank test. Survival data were censored at the time of re-irradiation or surgical intervention (including liver transplantation or resection). The univariable Cox proportional-hazards model was used to determine the predictors of IFC and OS. Factors with a *p*-value of <0.1 were included in the multivariable model, and a backward stepwise logistic regression model was used to avoid missing important confounders. Fisher's exact test was used to compare radiation-induced liver disease (RILD) events between CTP classes. Differences were considered statistically significant at a *p*-value of <0.05.

## Results

### Patients and treatment

This study included 89 patients. The median age was 68 years (range, 36–87 years), and 65 patients (73%) were men. Before SABR, most patients had underlying viral hepatitis predominantly related to hepatitis B virus [HBV] infection (46 patients, 51.7%), CTP class A liver function (69 patients, 77.5%), and American Joint Committee on Cancer stage of ≥III (69 patients, 77.5%). Other patient characteristics are summarized in Table 1.

**Table 1:** Patient and treatment characteristics.

		No.(%)
No. of patients		89 (100)
Sex	Male/Female	65 (73.0)/24 (27.0)
Age, years	Median/Range	68/36-87
Viral hepatitis	HBV	46 (51.7)
	HCV	28 (31.5)
	Both	3 (3.4)
	None	12 (13.5)
Recurrent tumor	Yes/ No	54 (60.7)/35(39.3)
Largest tumor size, cm	Median/Range	6.2/1.2–18.5
Sum of largest diameters of tumor, cm	Median/Range	6.6/1.5–23.5
Tumor number	Solitary/Multiple	31/58
Macrovascular invasion	Yes/No	44 (49.4)/45 (50.6)
mPVTT	Yes/No	23 (25.8)/66 (74.2)
Extrahepatic spread	Yes/No	26 (29.2)/63 (70.8)
TNM stage	I	7 (7.9)
	II	13 (14.6)
	IIIA/ IIIB/ IIIC	7 (7.9)/ 34 (38.2)/2 (2.2)
	IVA/ IVB	11 (12.4)/15 (16.9)
ECOG performance status	0–1	76 (85.4)
	2	13 (14.6)
AFP Level	< 400/≥ 400	49 (55.1)/40 (44.9)
CTP Classification	A/B	69 (77.5)/20 (22.5)
Combined systemic treatment	No	50 (56.2)
	Thalidomide	14 (15.7)
	Sorafenib	23 (25.8)
	Tegafur/Uracil	2 (2.2)

*Abbreviation*: SABR = stereotactic ablative radiotherapy; HBV = hepatitis B virus; HCV = hepatitis C virus; mPVTT = main portal vein tumor thrombosis; ECOG = Eastern Cooperative Oncology Group; AFP = α-fetoprotein; CTP = Child-Turcotte-Pugh liver function scale; TACE = Transarterial chemoembolization

Doses of 25–60 Gy in 4–6 fractions were prescribed to the 62–83% isodose curves. The median total dose and EQD2 were 45 Gy and 71.2 Gy, respectively. The most common regimen was 40 Gy in 5 fractions (19 patients), followed by 45 Gy (18 patients) or 50 Gy (14 patients) in 5 fractions.

### Responses

Follow-up CT or MRI data were available for 84 patients. The best primary tumor responses were CR in 22 patients (26.2%), partial response (PR) in 42 patients (50.0%), stable disease (SD) in 15 patients (17.9%), and PD in 5 patients (6.0%). The macrovascular thrombosis responses were CR in 2 patients, PR in 18, SD in 16, PD in 4, and not evaluable in 4. Of these patients, 3 received subsequent TACE.

Intrahepatic out-field recurrence, the main cause of treatment failure, was observed in 50 patients (59.5%). The 3-year IFC rate was 78.1%. There was no significant difference in IFC between patients with or without systemic treatment (no vs. any systemic treatment, *p* = 0.262; no vs. sorafenib, *p* = 0.410). The 3-year IFC rates were 72.5%, 88.6%, and 89.3% for patients treated with SABR alone, sorafenib, and any systemic treatment, respectively. No factors were identified as predictors of improved IFC in the univariable analysis.

### Downstaging and bridging to liver transplantation

Three patients underwent liver transplantation after SABR with doses of 35 Gy (1 tumor), 47.5 Gy (1 tumor), and 55 Gy (2 tumors in 1 patient). The first imaging evaluation after SABR revealed that all 3 patients had a PR, and downstaging and consideration for liver transplantation were approved by a multidisciplinary tumor board. The mean interval between SABR and liver transplantation was 3.4 months (range: 2.1–4.2 months). Explant pathology indicated a CR in 2 patients and PR in 1 patient after 55 Gy, and all 3 patients remained alive at the time of the analysis. The patient with pathological PR experienced a lung metastasis at 39 months after the liver transplantation. The 2 remaining patients did not exhibit evidence of recurrent HCC.

### Survival

**Fig 1:**
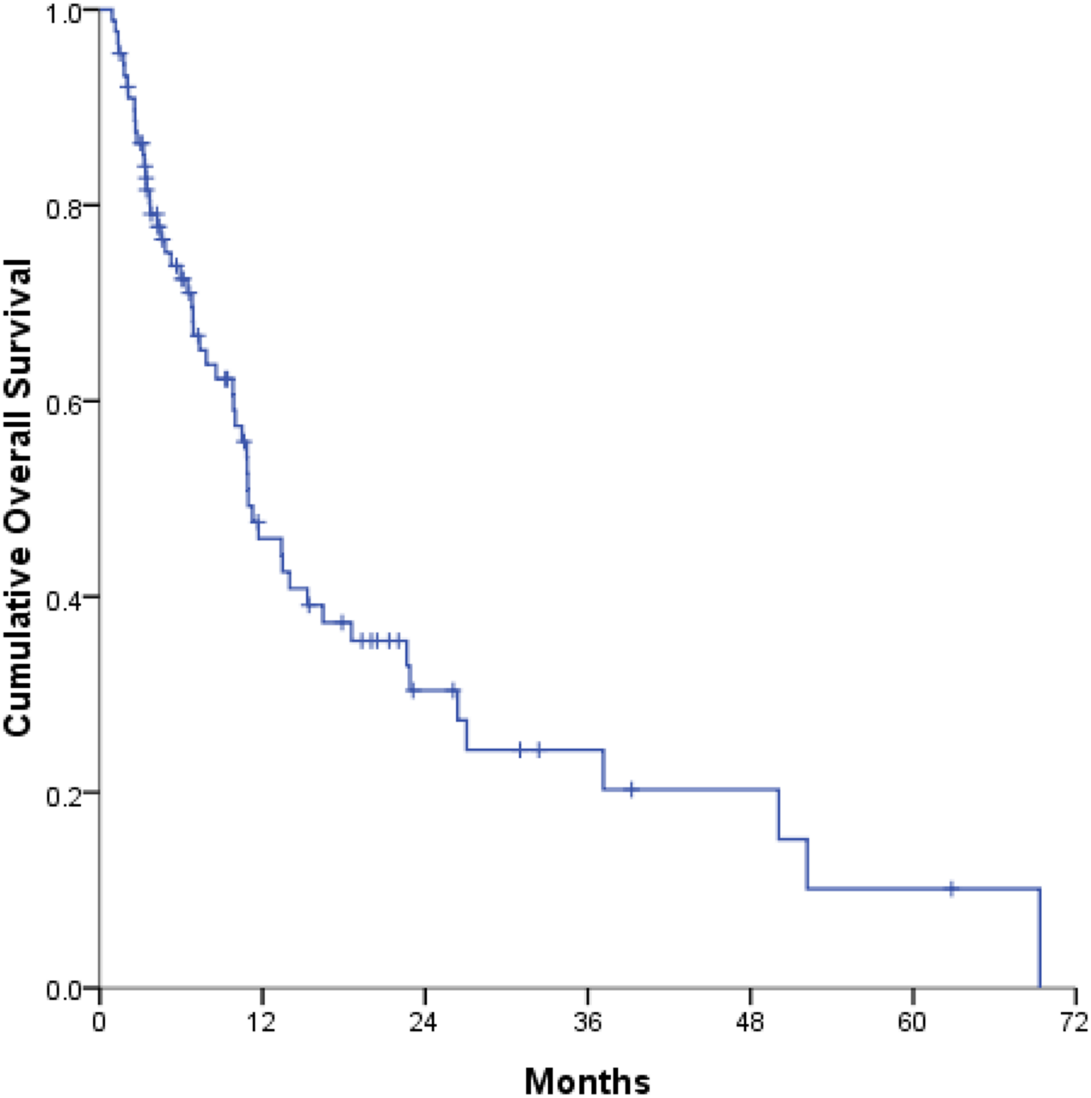
Kaplan–Meier curve of overall survival (OS). The 1-year and 3-year OS rates were 45.9% and 24.3%, respectively. The median OS time was 10.9 months.

At the time of the analysis, 54 patients had died and 35 patients remained alive. The median survival time was 10.9 months, and the 1-year, 3-year, and 5-year OS rates were 45.9%, 24.3%, and 10.1%, respectively (Fig 1). Univariable and multivariable analyses were performed to identify the parameters predictive of OS (Table 2). In the univariable analyses, several factors were significantly associated with OS, including the tumor number (multiple vs. solitary), sum of the largest tumor diameters (>6 cm vs. ≤6 cm), performance status (2 vs. 0–1), CTP classification (B vs. A), presence of main portal vein tumor thrombosis (mPVTT; yes vs. no), and presence of ES (yes vs. no).

**Table 2:** Prognostic factors on overall survival by cox proportional-hazards model.

Variables	Univariable		Multivariable	
	HR (95% CI)	*p*	HR (95% CI)	*p*
Age, years				
>60 vs. ≤60	0.67 (0.39–1.17)	0.158		
Sex				
Female vs. male	1.10 (0.61–1.98)	0.750		
Viral hepatitis				
HBV vs. no	1.87 (0.78–4.51)	0.162		
HCV vs. no	1.41 (0.54–3.68)	0.482		
Diagnosis at SABR				
Recurrent vs. New	0.71 (0.41–1.24)	0.232		
Tumor number				
Multiple vs. solitary	2.05 (1.12–3.76)	0.020		
Sum of largest diameters of tumor, cm				
>6 vs. ≤6	2.21 (1.20–4.08)	0.011		
mPVTT				
Yes vs. no	2.54 (1.42–4.54)	0.002	1.92 (1.03–3.59)	0.040
Extrahepatic spread				
Yes vs. no	2.92 (1.63–5.20)	<0.001	1.95 (1.00–3.79)	0.049
ECOG performance status				
2 vs. 0–1	2.08 (1.06–4.06)	0.033		
CTP classification				
B vs. A	5.59 (2.87–10.88)	<0.001	3.37 (1.56–7.27)	0.002
AFP level				
≥400 vs. <400	1.26 (0.74–2.17)	0.396		
Combined systemic treatment				
Yes vs. no	1.67 (0.97–2.87)	0.065		
Total dose, Gy				
>40 vs. ≤40	0.89 (0.51–1.55)	0.685		
EQD2, Gy				
>66 vs. ≤66	1.08 (0.63–1.86)	0.783		

Abbreviation: HR = hazard ratio; HBV = hepatitis B virus; HCV = hepatitis C virus; SABR = stereotactic ablative radiotherapy; mPVTT = main portal vein tumor thrombosis; ECOG = Eastern Cooperative Oncology Group; CTP = Child-Turcotte-Pugh liver function scale; AFP = α-fetoprotein; EQD2 = equivalent dose of 2 Gy per fraction

**Fig 2:**
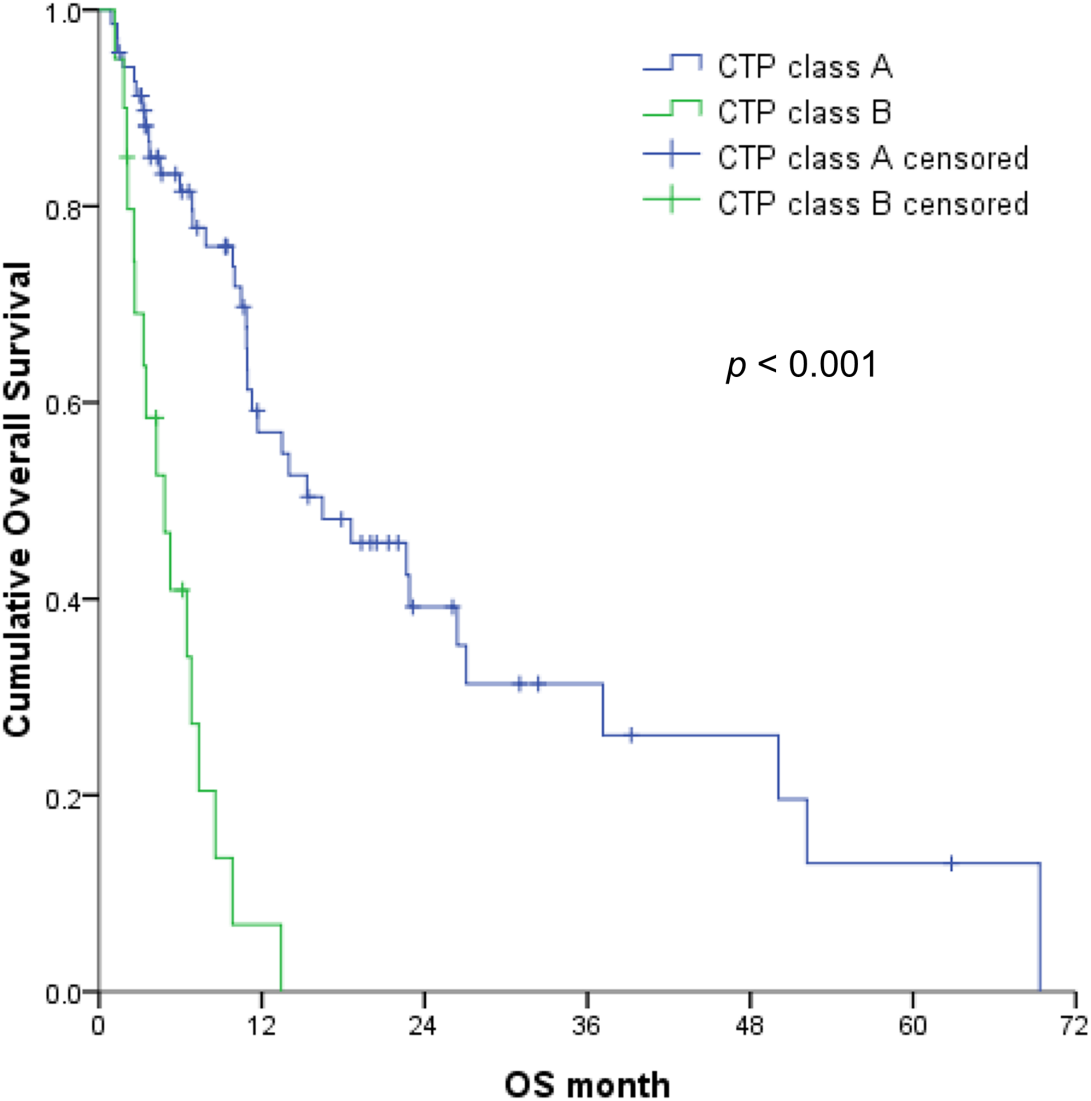
Overall survival of hepatocellular carcinoma patients with Child–Turcotte–Pugh class B vs. class A liver function (1-year OS, 6.8% vs. 57%).

**Fig 3:**
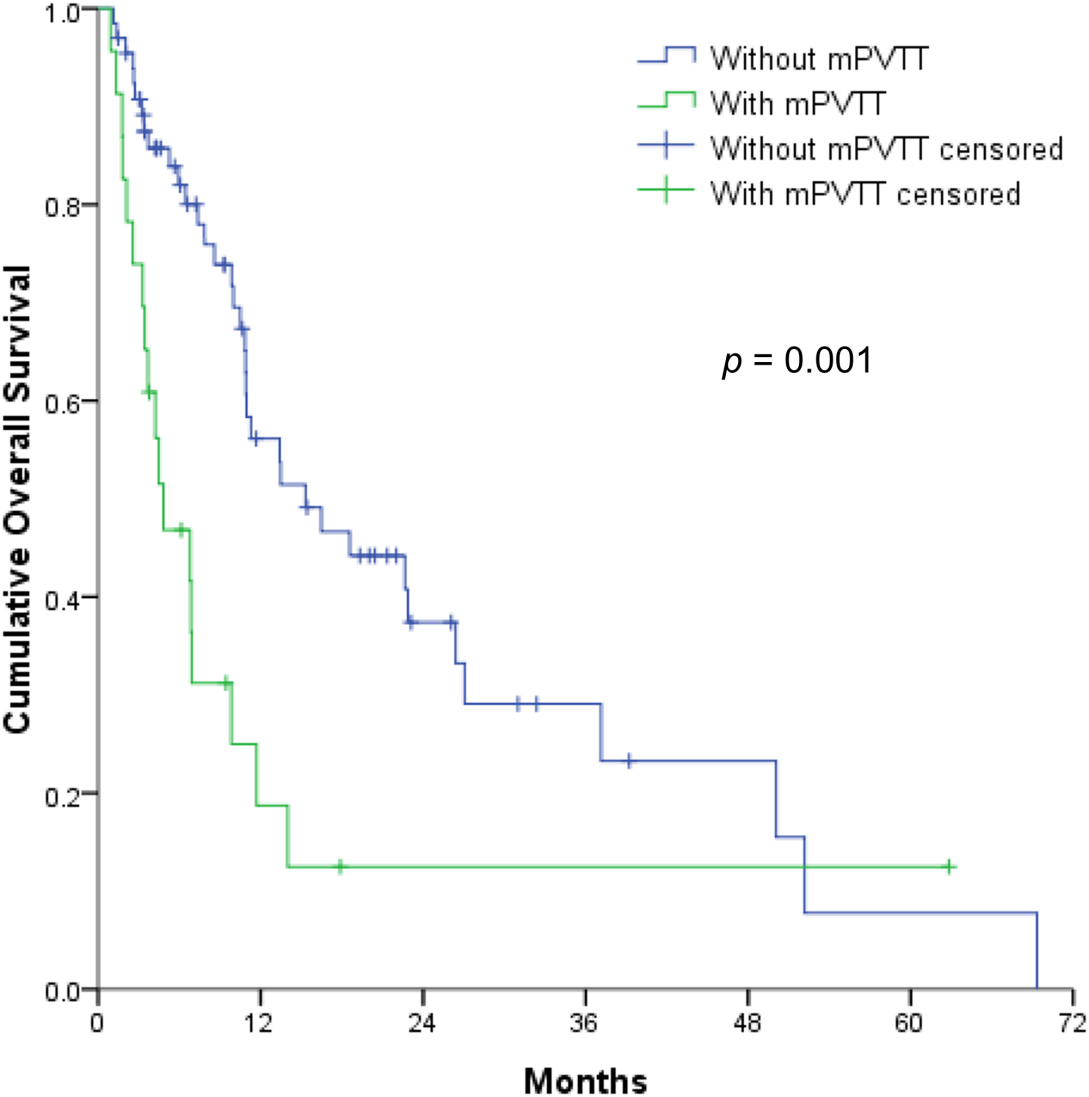
Overall survival of hepatocellular carcinoma patients with main portal vein tumor thrombosis (mPVTT) vs. those without mPVTT (1-year OS, 18.7% vs. 56.1%).

**Fig 4:**
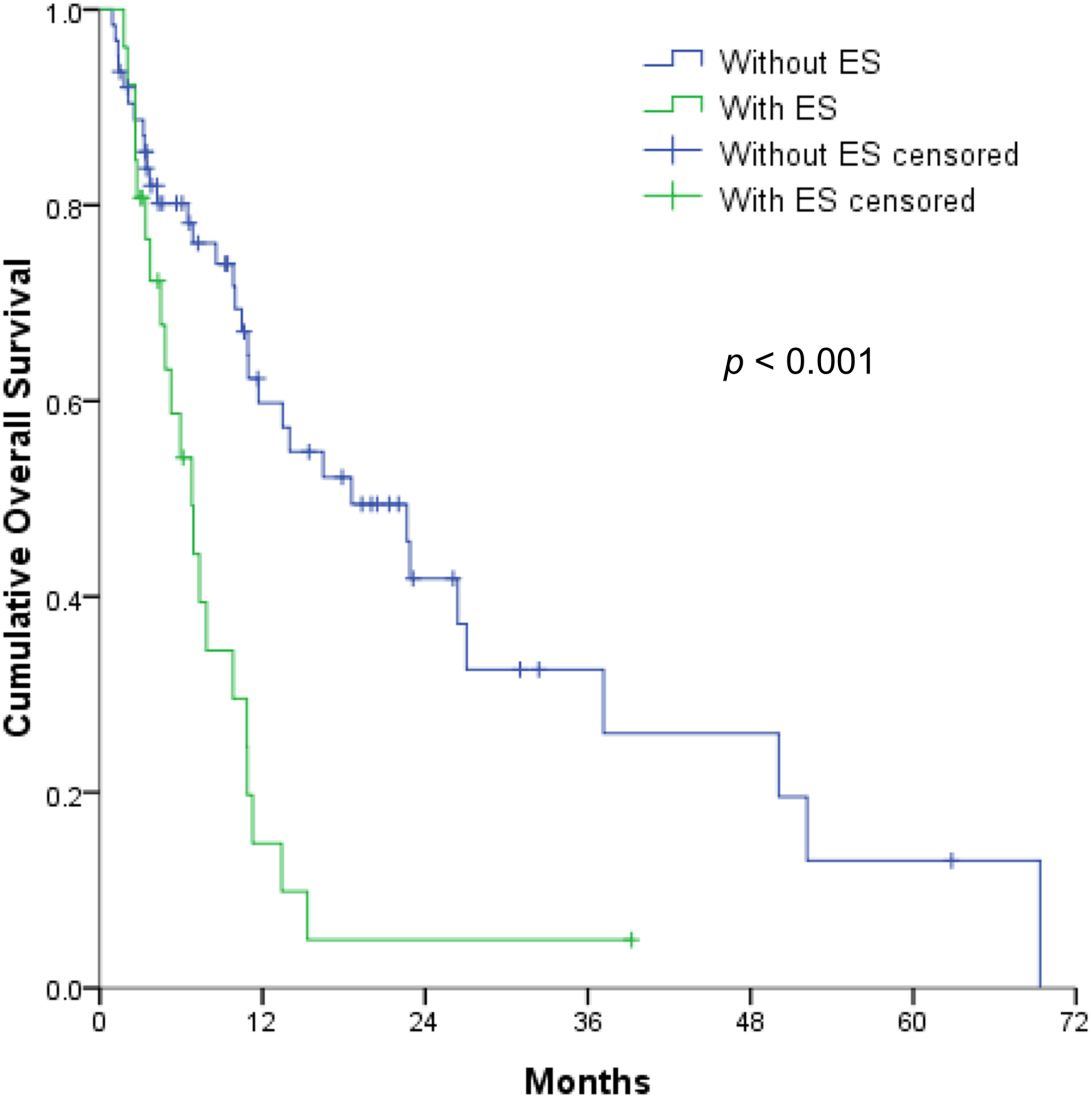
Overall survival of hepatocellular carcinoma patients with extrahepatic spread (ES) vs. patients without ES (1-year OS, 14.8% vs. 59.8%).

**Fig 5:**
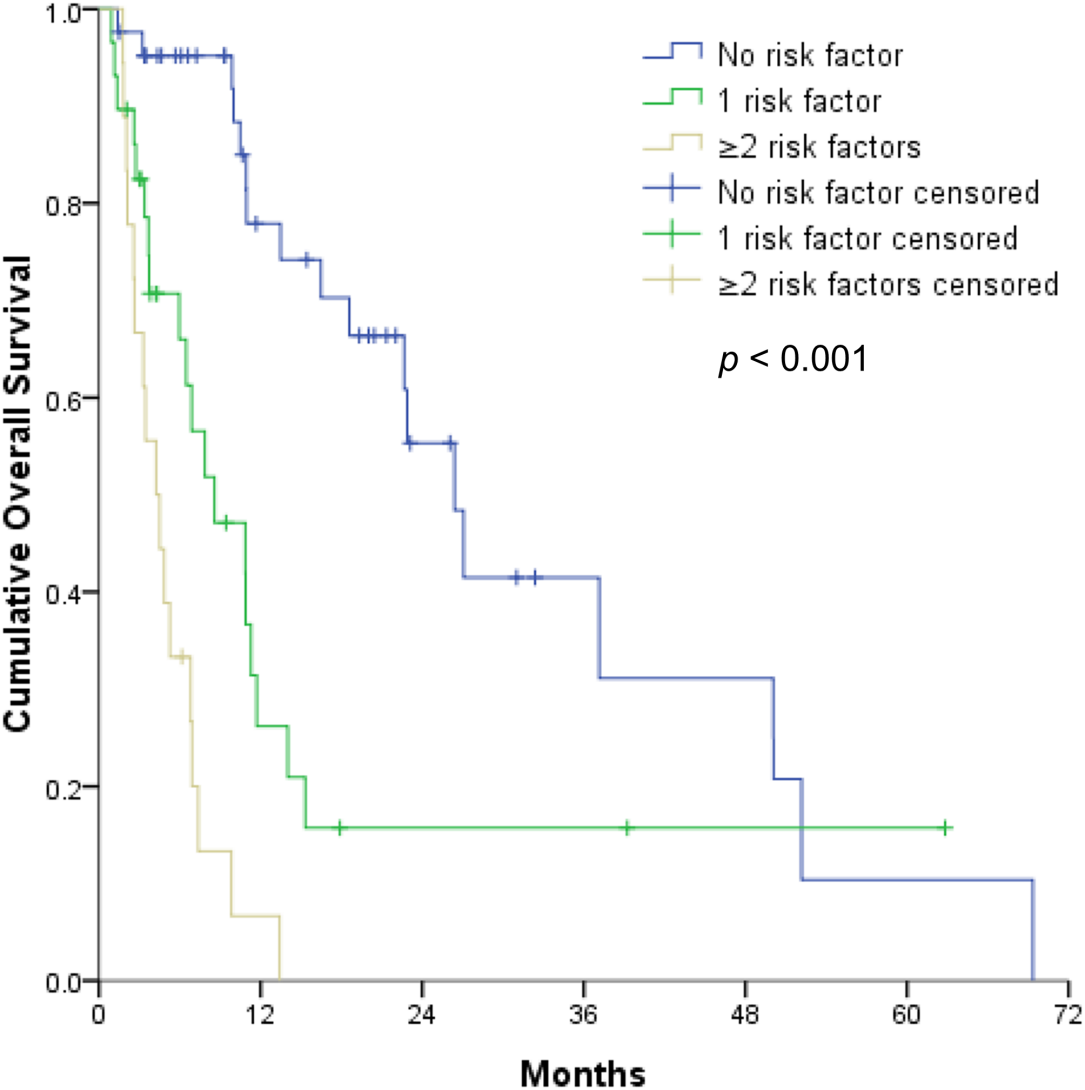
Kaplan–Meier curve of overall survival (OS) as a function of the number of risk factors among hepatocellular carcinoma patients stratified by Child–Turcotte–Pugh classification, main portal vein tumor thrombosis, and extrahepatic spread. The 1-year OS rates among patients with 0, 1 and ≥ 2 risk factors were 77.9%, 26.2%, and 6.7%, respectively.

The multivariable analyses revealed that OS was independently associated with the CTP classification (Fig 2), mPVTT (Fig 3), and ES (Fig 4) (all *p* < 0.05). Therefore, the patients were divided into 3 subgroups according to the number of risk factors (no risk factors, n = 42; 1 risk factor, n = 29; ≥2 risk factors, n = 18). OS significantly decreased as the number of risk factors increased (*p* <0.001) (Fig 5), with expected median OS values of 4.2 months, 8.6 months, and 26.4 months for ≥2 risk factors, 1 risk factor, and 0 risk factors, respectively.

Patients who were also treated with sorafenib had a median survival of 9.9 months, which was inferior to the interval of 13.5 months for patients without any systemic treatment (*p* = 0.049). Sorafenib use was included in the multivariable analysis but was not found to be an independent predictor of survival. Furthermore, patients who responded to SABR had a significantly longer survival time, compared to those who did not respond (15.3 months vs. 7.4 months, *p* <0.001).

### Toxicity

All patients received the planned radiotherapy without interruption due to SABR-related toxicity. The acute toxicities are described in Table 3. SABR was generally tolerable, with no grade 4 or higher toxicities (other than RILD). Fatigue, abdominal pain, nausea/vomiting, and anorexia were the most common adverse effects.

**Table 3:** Acute toxicities.

Toxicity	No. of patients (%)		
	Grade 1	Grade 2	Grade 3
Fatigue	22 (24.7)	4 (4.5)	0
Anorexia	12 (13.5)	2 (2.2)	0
Nausea/Vomiting	12 (13.5)	11 (12.4)	1(1.1)
Abdominal distension	4 (4.5)	0	0
Abdominal pain	17 (19.1)	7 (7.9)	2(2.2)
Gastritis/ gastric ulcer	0	3 (3.4)	2(2.2)
Duodenal ulcer	2 (2.2)	4 (4.5)	0
Diarrhea	1 (1.1)	2 (2.2)	0
Dermatitis	1 (1.1)	2 (2.2)	0

After excluding patients with PD, 32 and 13 patients exhibited decreases in CTP scores and classifications within 6 months, respectively. The magnitudes of the CTP score decreases were 1 point, 2–4 points, and ≥5 points in 20, 8, and 4 patients, respectively, and 13 patients achieved their baseline CTP score after conservative care. Ten patients (11.2%) developed RILD: 1 developed classic RILD, 8 developed non-classic RILD, and 1 fulfilled the criteria for both types. RILD was more common among patients with CTP class B vs. CTP class A liver function (5/69 vs. 5/20; *p* = 0.027)

Most patients with RILD recovered within 6 months after receiving supportive treatment, although 2 patients developed fatal non-classic RILD. The first patient was a 39-year-old male HBV carrier with newly diagnosed HCC and CTP class A liver cirrhosis. The patient underwent SABR without receiving prophylactic anti-HBV therapy. A dose of 50 Gy in 5 fractions was delivered to the bilateral branches and mPVTT lesion, and 1,173 cc of the normal liver received <15 Gy. At 3.5 weeks after completing SABR, the patient exhibited a CTP score deterioration (from A5 to B10) and markedly elevated serum transaminase levels, compatible with non-classic RILD. High serum HBV DNA levels (5,130,000 copies/mL) were also observed, suggesting HBV reactivation. The other patient was a 45-year-old man with recurrent HCC that had invaded the left branch and main portal vein. He had received anti-viral therapy for 1 year to treat chronic hepatitis B. This patient underwent salvage SABR with a dose of 40 Gy in 5 fractions, and 966 cc of the normal liver received <15 Gy. He was ultimately diagnosed with non-classic RILD and acute liver decompensation (CTP score reduction from A5 to B13) at 3 weeks after the completion of SABR.

## Discussion

The present study provides the largest analysis of SABR for patients with BCLC stage C HCC. Various treatment options are available for these patients, and previous studies have evaluated sorafenib, surgical resection, TACE, hepatic arterial infusion chemotherapy, radiotherapy, and multimodality management [5, 7, 18, 23–26]. The wide variance in median survival times of patients with HCC (1.9–27.8 months) is likely related to the heterogeneous spectrum of BCLC stage C disease and the inclusion criteria of the different studies. The best outcomes are typically achieved with surgery, and Yang *et al*. revealed through a large retrospective study that surgical resection for BCLC stage C HCC provided a median OS of 27.8 months (1-year, 3-year, and 5-year OS rates of 69.9%, 41.2%, and 30.5%, respectively) [23]. However, among the 511 patients in that study, only 63 (12.3%) had extrahepatic disease and 314 (61.4%) were included solely because of symptomatic disease. Torzilli *et al*. also evaluated the long-term outcomes of surgery for HCC in a multi-center study and reported 1-year, 3-year, and 5-year OS rates of 76%, 49%, and 38%, respectively [25]. However, their study only included patients with liver-restricted diseases, and 297 (14%) were classified as having BCLC stage C macrovascular disease. These promising outcomes are clearly favorable when compared with best supportive care or sorafenib treatment, highlighting the importance of local control for selected patients with advanced HCC.

Increasing evidence has led to the acceptance of SABR as a management strategy for HCC. SABR can be used as an ablative therapy in patients with early-stage unresectable tumors or a medically inoperable status, and yields good responses and high local control rates [8, 10]. Compared with RFA, SABR provides similar local control of small lesions (< 2 cm) but a better performance in larger lesions [27]. Regarding locally advanced disease, Bujold *et al*. performed the largest prospective SABR trial (24–54 Gy in 6 fractions) [17]. That study evaluated 102 patients with CTP class A function, BCLC stage C disease (67 patients, 65.7%), and intrahepatic disease (90 patients, 88.2%), and obtained 1-year local control and OS rates of 87% and 55%, respectively (median OS: 17.0 months). Another retrospective analysis of 35 patients with BCLC stage C disease (CTP class A function, 32 patients) yielded similar outcomes with varied doses and fractionation (30–60 Gy in 3–5 fractions) [18]. In that study, the median OS was 14 months, and the 1-year local control and OS rates were 69% and 52%, respectively. In the present study, the median survival time was 10.9 months, and the 1-year IFC and OS rates were 78.1% and 45.9%, respectively. These results were comparable with published SABR results, despite our inclusion of more cases with CTP class B function (22.5%) and ES (29.2%). Therefore, it may be appropriate to consider SABR during treatment selection for patients with BCLC stage C HCC, especially for patients who are not suitable for sorafenib treatment.

In the present study, the multivariable analyses identified three factors that independently predicted OS after SABR. Two of these factors were related to the tumor status, and 1 factor was related to the underlying liver function, thus confirming the reliability of the BCLC classification. In addition, the findings of the present study and previous studies confirm that CTP class is a clear prognostic factor [2, 4, 6]. For example, Bae *et al*. demonstrated that the CTP class was the most significant prognostic factor among patients with BCLC stage C disease after SABR, with 1-year survival rates of 69% and 0% for CTP class A and class B, respectively [18]. In another small study, Culleton *et al*. evaluated 28 patients with HCC after SABR (20 patients, 69% with CTP B7 function and most with BCLC stage C disease), and found that a CTP score of ≥8 was significantly associated with poorer survival [15]. Therefore, based on the available evidence, caution should be exercised when selecting SABR for patients with CTP class B function.

Several factors related to the HCC tumor status are important survival determinants of survival and have been used for cancer staging, despite the lack of consensus regarding a favorable HCC tumor status among the published SABR studies. For example, tumor size has been proposed as an independent prognostic factor for OS after SABR [13–15]. Moreover, tumor vascular thrombosis was significantly associated with OS [17]. However, in the present study, we found that the presence of mPVTT and ES were independent prognostic factors for OS. Therefore, it is difficult to draw firm conclusions regarding the optimal tumor status for SABR, given the diverse study designs and patient characteristics.

In our opinion, further prognostic stratification based on pretreatment factors (e.g., tumor status and liver function) could facilitate the appropriate management of patients with HCC. This strategy might help to identify the best candidates for SABR among the broad spectrum of patients with BCLC stage C disease. In the present study, the median OS among patients with ≥2 risk factors, 1 risk factor, and 0 risk factors were 4.2 months, 8.6 months, and 26.4 months, respectively. Besides, a nearly 8-month gain in OS was observed among those who responded to SABR. Therefore, our results highlight the importance of local control in selected patients with advanced HCC and warrant the incorporation of SABR in BCLC system treatment algorithms.

The toxicity profile in the present study was acceptable and similar to profiles reported from previous studies [9, 10, 12, 13, 17, 18]. In the present study, grade ≥3 toxicities were observed in 15 patients (16.9%), including 10 cases of RILD. Furthermore, 12 patients experienced a decline in the CTP score of ≥2 points within 6 months after SABR. However, the other toxicities were generally mild and self-limited, and this liver-centric toxicity profile is compatible with the precise delivery of SABR. Interestingly, RILD has been significantly associated with the HBV status and CTP class B liver cirrhosis [28], and several studies have adopted stricter dose volume constraints and/or dose modifications according to liver function [10, 15, 16, 19]. The present study further revealed that RILD was more common among patients with CTP class B vs. CTP class A liver cirrhosis (5/69 vs. 5/20; *p* = 0.027). Unfortunately, 1 patient died from HBV reactivation-related RILD within 1 month after SABR, despite exhibiting favorable characteristics at the dosimetry planning. These findings therefore highlight the importance of strict patient selection and personalized treatment in cases of HCC.

The major limitation of this study was the single-center retrospective design, which might have led to selection bias. Nevertheless, this was the largest study of SABR for advanced HCC according to the BCLC system. Therefore, despite the limitations, the results warrant a prospective trial. Furthermore, this study revealed that SABR provided acceptable toxicity, sustained local control, and favorable survival benefits for selected patients with advanced HCC. Moreover, the CTP class, mPVTT, and ES were independent predictors of OS and may therefore be useful when selecting patients to undergo SABR for HCC.
